# Supplementary material for: The genomic landscape of cholangiocarcinoma reveals the disruption of post-transcriptional modifiers
Source: Nat Commun. 2022 Jun 1;13:3061. doi: 10.1038/s41467-022-30708-7 (PMC9160072; doi:10.1038/s41467-022-30708-7)
Supplement: Supplementary file 1 — Supplementary Information [file 41467_2022_30708_MOESM1_ESM.pdf]

The genomic landscape of cholangiocarcinoma reveals the disruption of post-transcriptional modifiers

Supplementary Figure 1

Supplementary Figure 2

Supplementary Figure 3

Supplementary Figure 4

Supplementary Figure 5

Supplementary Figure 6

Supplementary Figure 7

Supplementary Table 1

Supplementary Table 2

Supplementary Table 3

Supplementary Table 4

Supplementary Table 5

Supplementary Table 6

Supplementary Table 7

Supplementary Table 8

Supplementary Table 9

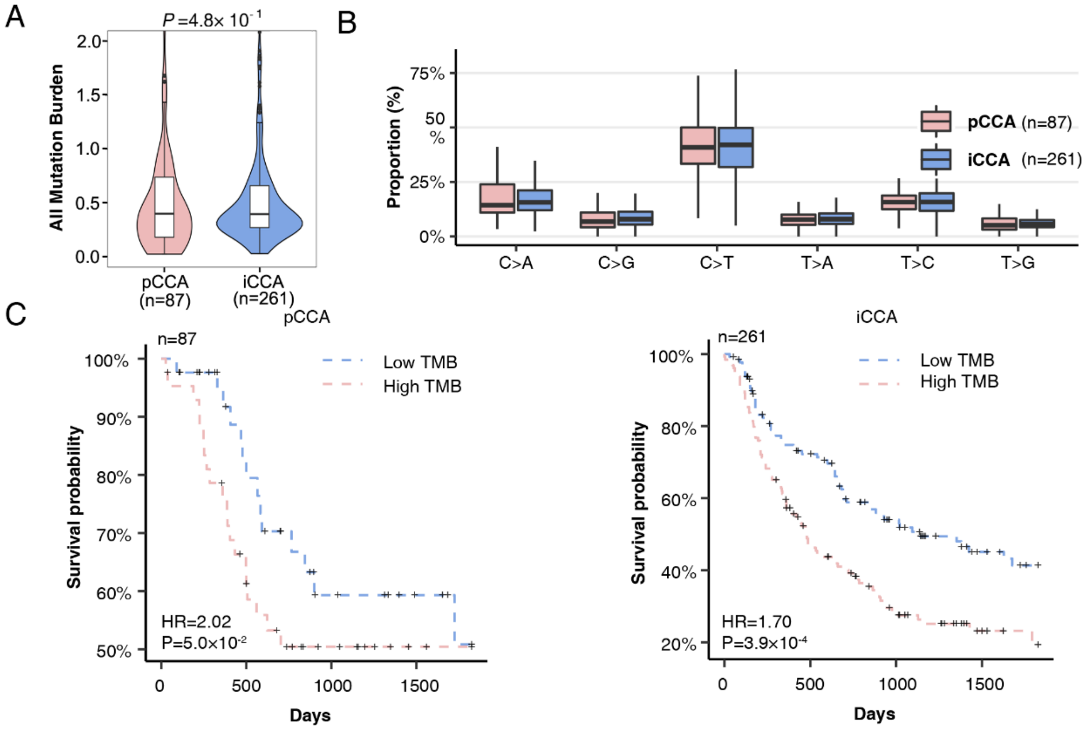

Supplementary.Fig.1 **(A)** Boxplot of all mutation burden in pCCA and iCCA. Wilcoxon rank-sum test was performed to obtain the P value. **(B)** Constitution of six types of single nucleotide substitutions between iCCAs (presented in blue) and pCCAs (presented in light red). Box plots depicted the median, quartiles and range. The whiskers in box plots extended to the most extreme data point which is no more than 1.5 times IQR. Outliers were identified using upper/lower quartile ±1.5 times IQR. **(C)** Kaplan-Meier survival plot between iCCA (left panel) and pCCA (right panel) patients with high TMB and low TMB. Cox proportional hazards model adjusted for age, gender, and tumor stage was performed to obtain HR and P values.

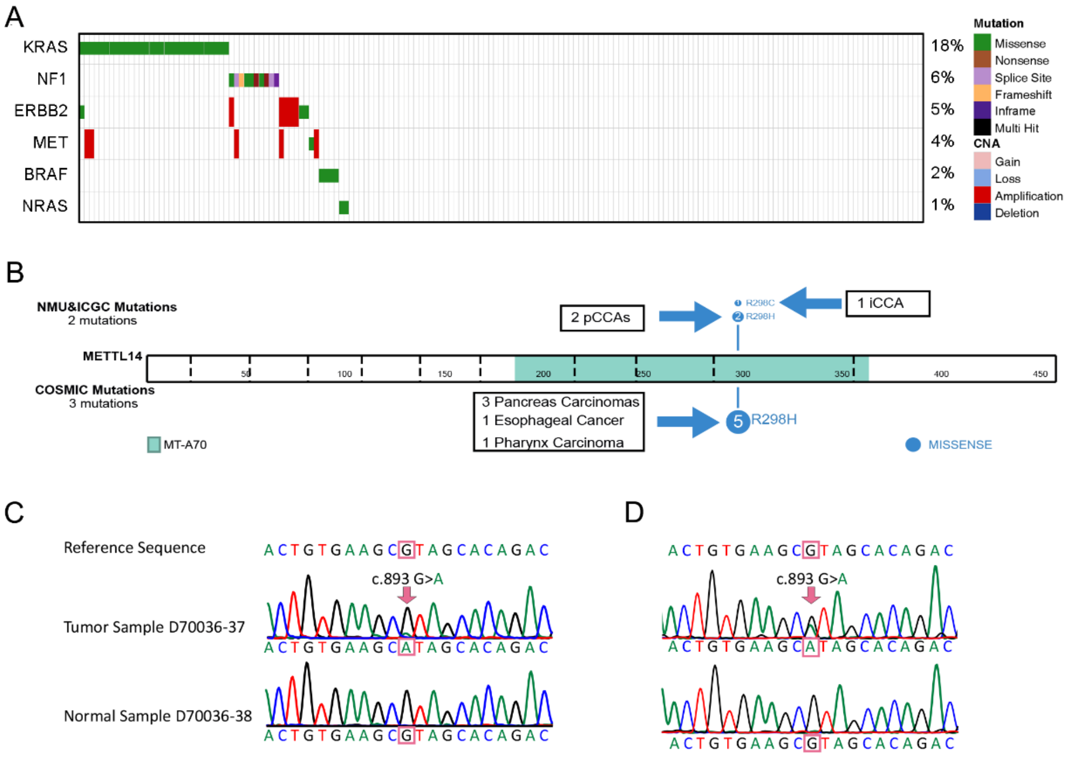

Supplementary.Fig.2 (A) Characters in CNA and mutation of driver genes in RAS-RTK pathway. **(B)** Lolliplot of METTL14 R298H and R298C. The top panel represents mutations in this study and the bottom panel represents mutations from COSMIC dataset. **(C & D)** Sanger sequencing plot of interested region including METTL14 R298H of the NMU subject **(C)** and the additional subject (**D**).

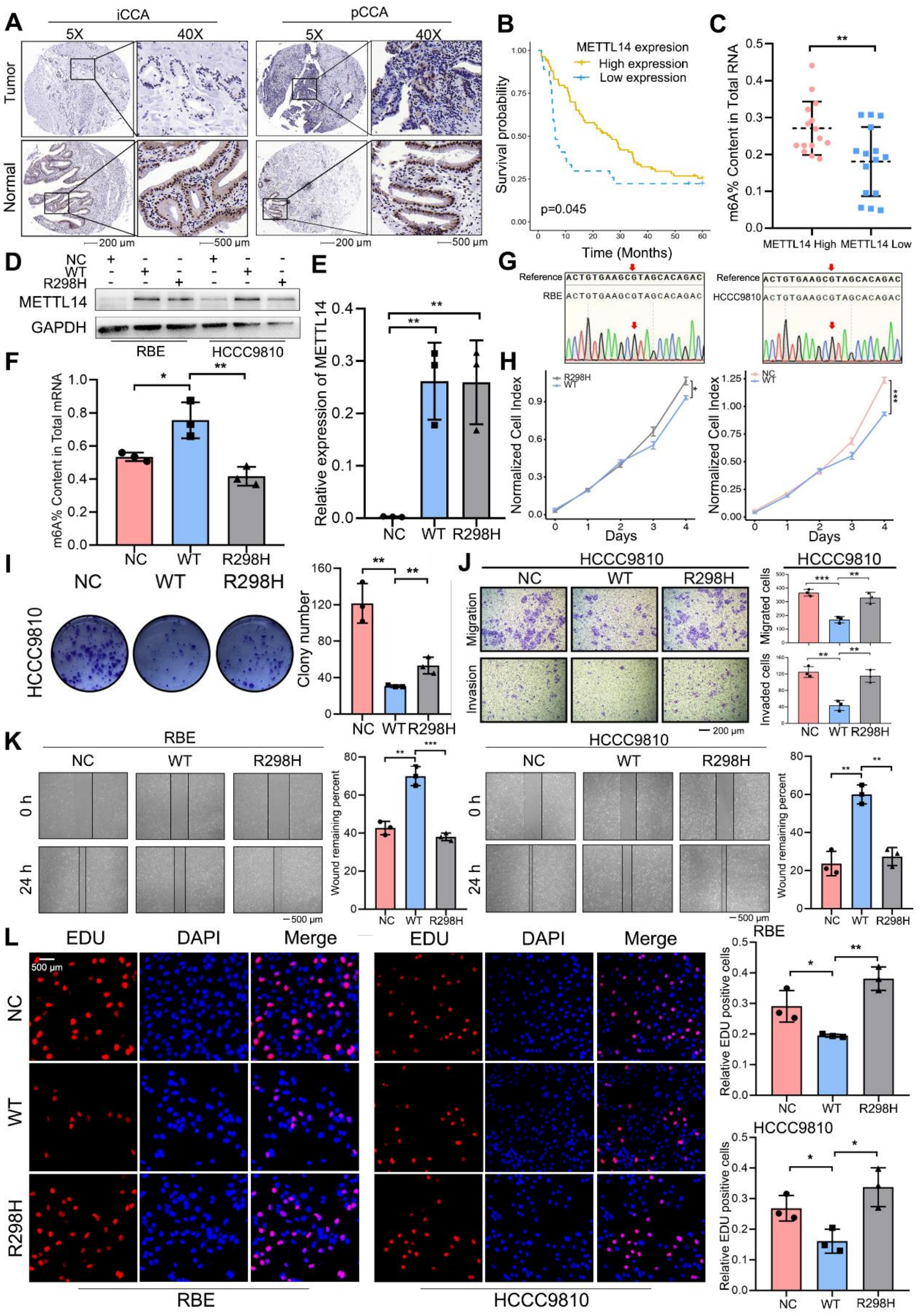

Supplementary.Fig.3 (A) Representative IHC stains of METTL14 in iCCA and pCCA tissues and matched adjacent normal tissues. **(B)** METTL14 downregulation in CCA tissues was associated with shorter cancer-specific survival in CCA patients (n=111).

(C) The m^6A contents of total mRNA in METTL14 High group (n=15) and METTL14 Low group (n=15). **(D)** Stable $METTL14^{R298H}$ and $METTL14^{wt}$ Cells were screened by western blot. **(E)** The transfection efficiency of lentiviral constructs expressing $METTL14^{wt}$ and $METTL14^{R298H}$ in HCCC9810 cell line (n=3). **(F)** $METTL14^{R298H}$ reduced $METTL14^{wt}$-mediated m^6A modification detected by m^6A colorimetric quantification in HCCC9810 cell line (n=3). **(G)** Sanger sequencing plot of interested region including METTL14 R298 of cholangiocarcinoma cell lines (RBE and HCCC9810). **(H)** Proliferation curve of HCCC9810 cells with $METTL14^{R298H}$, $METTL14^{wt}$, or negative control (n=3). **(I)** Colony formation assay of HCCC9810 cells with $METTL14^{R298H}$, $METTL14^{wt}$, or negative control. The number of colonies were counted and presented in the histogram (n=3). **(J)** Representative images (left) and quantification (right) of transwell migration and invasion assays in HCCC9810 cells with $METTL14^{wt}$, $METTL14^{R298H}$, or negative control (n=3). **(K)** Wound healing assay in RBE and HCCC9810 cells with $METTL14^{R298H}$, $METTL14^{wt}$, or negative control (n=3). **(L)** Representative images (left) and quantification (right) of EDU assays in RBE and HCCC9810 cells with $METTL14^{wt}$, $METTL14^{R298H}$, or negative control (n=3). The P values were calculated using unpaired two-sided Student's t test with no correction for multiple comparison. Data are shown as mean ± SEM. $*P < 0.05$, $**P < 0.01$, $***P < 0.001$; R298H, $METTL14^{R298H}$; WT, $METTL14^{wt}$; NC, negative control.

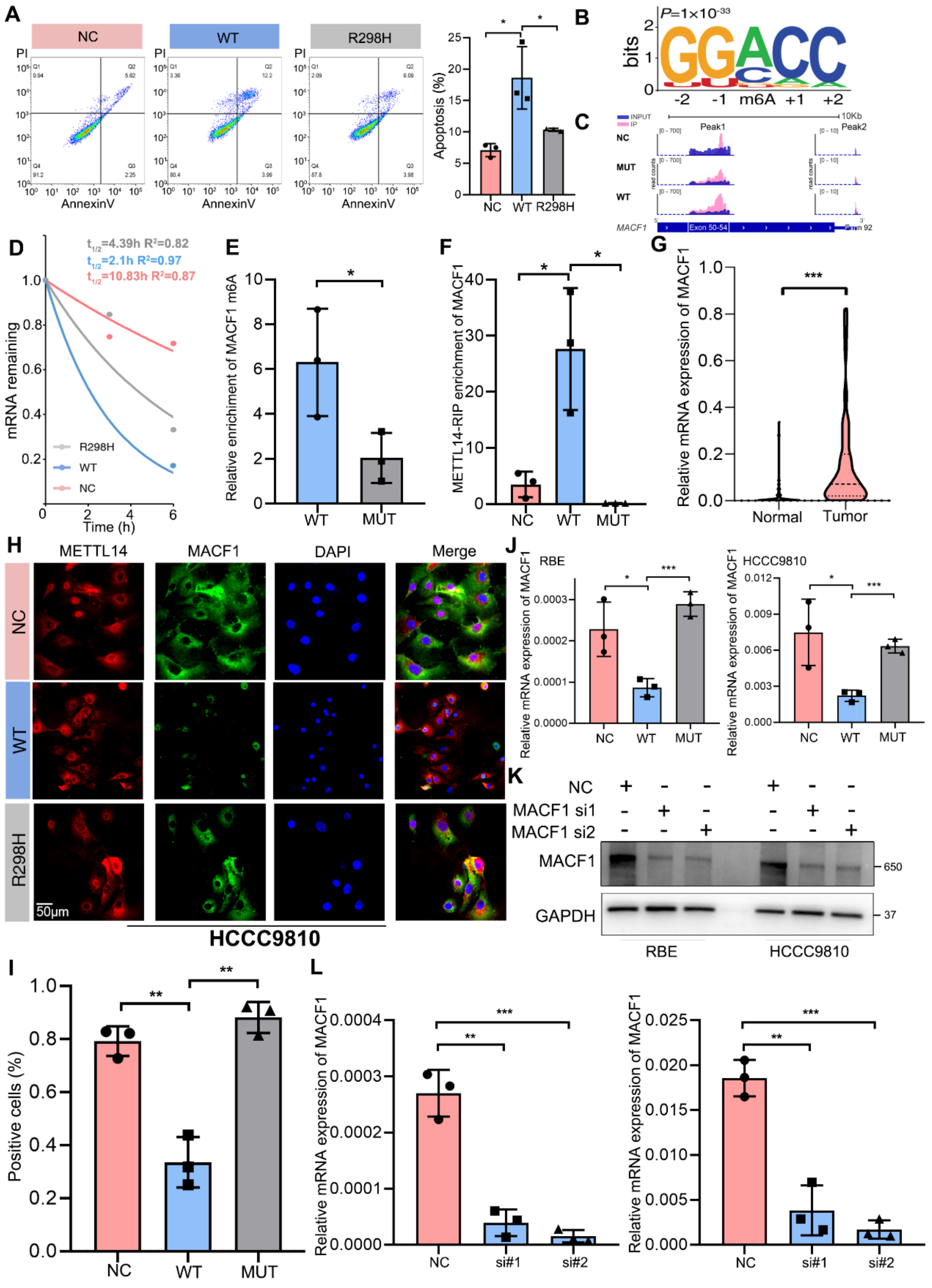

Supplementary.Fig.4 (A) Apoptotic assay of HCCC9810 cells with METTL14^{R298H}, METTL14^{wt}, or negative control were determined by a PI and annexin V double-staining assay and analysis by flow cytometry (n=3). **(B)** GGAC is the most common m^{6}A motif significantly enriched in the m^{6}A peaks, and the m^{6}A peaks are especially

enriched in the vicinity of the stop codon. **(C)** Gene plots of MACF1 coding region harboring m6A peaks. Coverage of IP and input control is indicated in red and blue, respectively. The blue boxes in the bottom panel represent exons and UTRs. **(D)** RNA lifetime for MACF1 in HCCC9810 cells transfected with METTL14^{R298H}, METTL14^{wt}, or negative control. **(E)** The m^{6}A modification level of MACF1 was validated in methylated RNA Immunoprecipitation (MeRIP) (n=3). **(F)** Immunoprecipitation of METTL14-related RNA in NC, METTL14^{wt} and METTL14^{R298H} was conducted followed by RT-qPCR to detect the amount of MACF1 mRNA binding to METTL14 (n=3). **(G)** Upregulated MACF1 mRNA expression was detected in 66 pairs of CCA tumor tissues by qRT-PCR (p<0.001). **(H)** Representative images of MACF1 immunofluorescence in METTL14^{wt} and METTL14^{R298H} containing in HCCC9810 cells. All data are representative of at least two independent experiments with similar results. **(I)** The bar plot shows the MACF1 foci number in the METTL14 positive cells (n=3). **(J)** MACF1 mRNA expression in METTL14^{wt} and METTL14^{R298H} in RBE and HCCC9810 cell lines (n=3). **(K)** The knockdown efficiency of MACF1 in RBE and HCCC9810 cell lines was tested by western blot analysis. **(L)** Knockdown of MACF1 in RBE and HCCC9810 cells by siRNA were verified by qRT-PCR (n=3). The P values were calculated using unpaired two-sided Student's t test with no correction for multiple comparison. Data are shown as mean ± SEM. *P < 0.05, **P < 0.01, ***P < 0.001; R298H, METTL14^{R298H}; WT, METTL14^{wt}; NC, negative control.

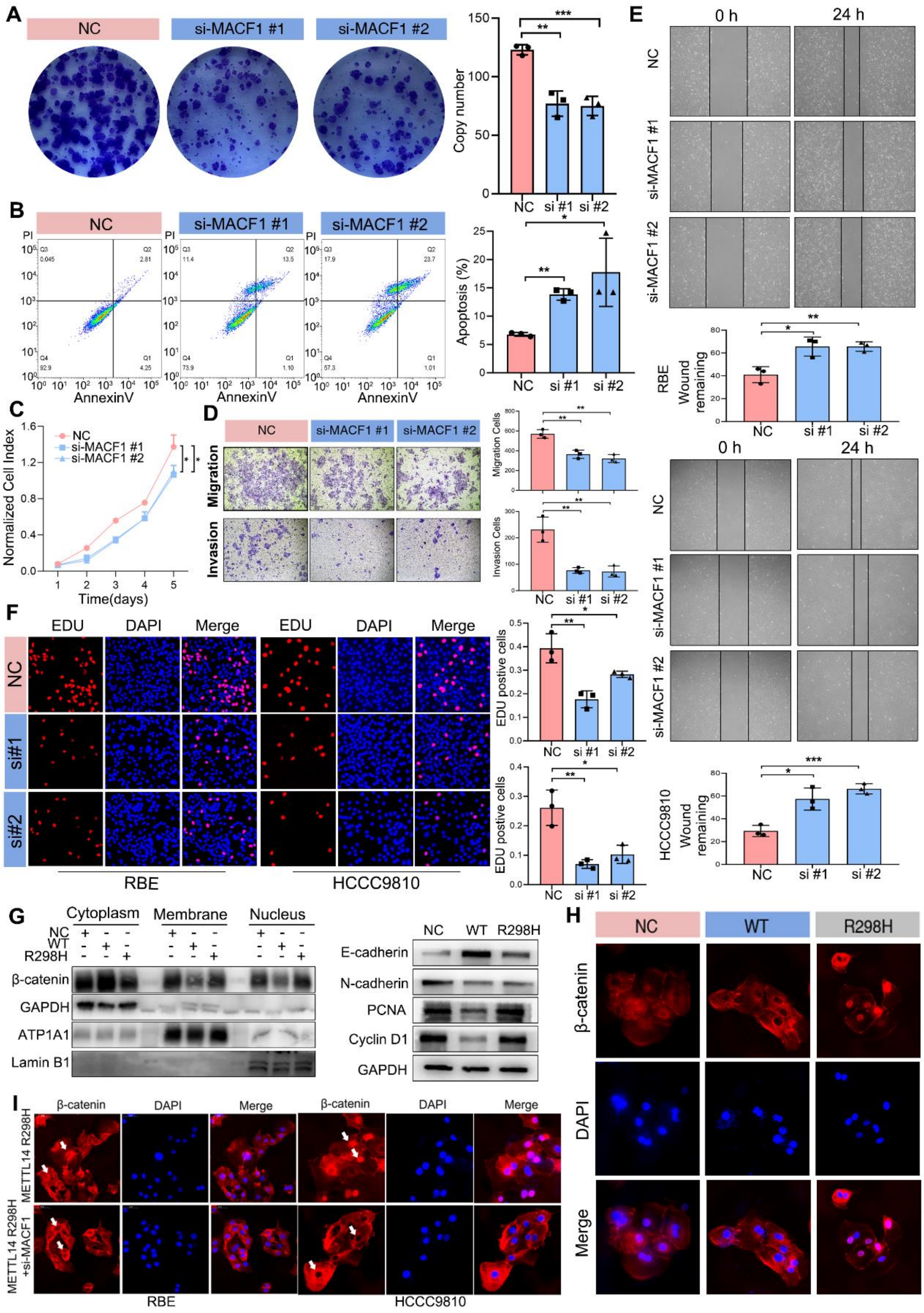

Supplementary.Fig.5 (A) Colony formation assay of HCCC9810 cells with negative control and MACF1-siRNA (n=3). **(B)** Apoptotic assay of HCCC9810 cells with negative control and MACF1-siRNA were determined by a PI and annexin V double-staining assay and analysis by flow cytometry (n=3). **(C)** Proliferation curve of

HCCC9810 cells with negative control and MACF1-siRNA (n=3). **(D)** Representative images (left) and quantification (right) of transwell migration and invasion assays in HCCC9810 cells with negative control and MACF1-siRNA (n=3). **(E)** Wound healing assay in RBE and HCCC9810 cells with negative control and MACF1-siRNA (n=3). **(F)** Representative images (left) and quantification (right) of EDU assays in RBE and HCCC9810 cells with negative control and MACF1-siRNA (n=3). **(G)** Western blot analysis was performed to analyze the expression of β-catenin in the cytoplasmic, membrane and nuclear extracts in HCCC9810 cells. All data are representative of at least two independent experiments with similar results. **(H)** Representative images of β-catenin immunofluorescence showed nuclear β-catenin tended to be increased by expression of $METTL14^{R298H}$ compared to $METTL14^{wt}$ in HCCC9810 cells. **(I)** MACF1 siRNA transfected in $METTL14^{R298H}$-overexpressing cells, and the representative images of nucleus translocation of β-catenin is shown using immunofluorescence. The P values were calculated using unpaired two-sided Student's t test with no correction for multiple comparison. Data are shown as mean ± SEM. *P < 0.05, **P < 0.01, ***P < 0.001; R298H, $METTL14^{R298H}$; WT, $METTL14^{wt}$; NC, negative control.

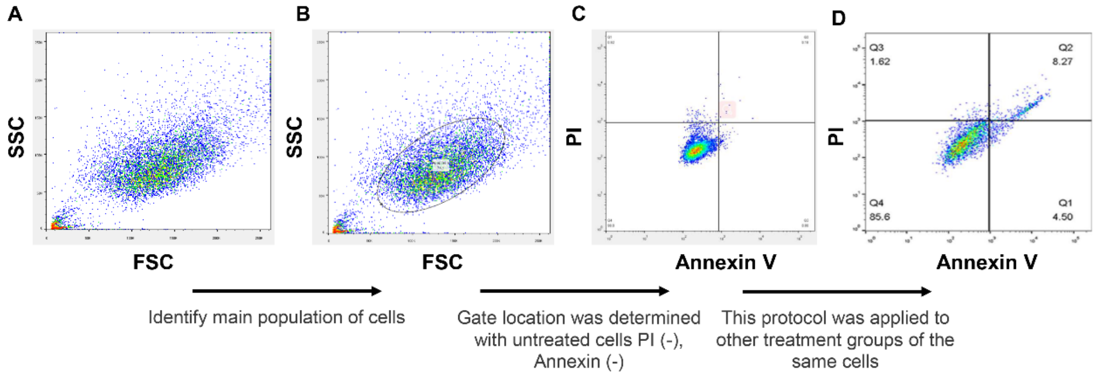

Supplementary.Fig.6 FACS Gating strategy for tumor cells

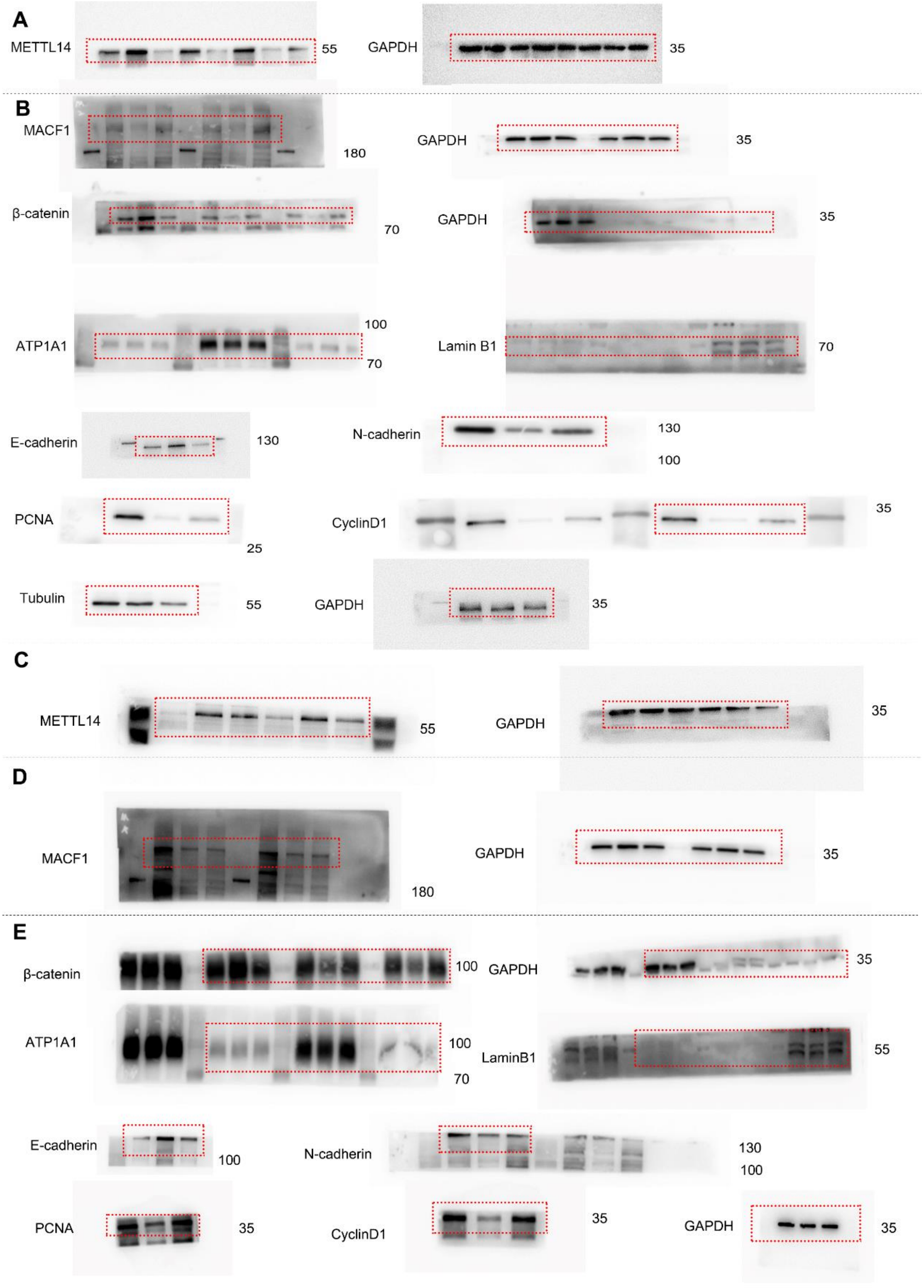

Supplementary.Fig.7 (A) Uncropped scans from Fig. 4. (B) Uncropped scans from Fig.5. (C) Uncropped scans from Supplementary Fig. 3. (D) Uncropped scans from Supplementary Fig. 4. (E)Uncropped scans from Supplementary Fig. 5.

Supplementary Table 1. General Description of Patients with CCA from Nanjing Medical University

	NMU		
	Total	iCCA	pCCA
Age			
Mean (SD)	61.0 (±9.3)	60.1 (±9.0)	61.5 (±9.6)
Gender			
Male	45 (67.2%)	16 (66.7%)	29 (67.4%)
Female	22 (32.8%)	8 (33.3%)	14 (32.6%)
HBV			
Yes	15 (22.4%)	8 (33.3%)	7 (16.3%)
No	51 (76.1%)	16 (66.7%)	35 (81.4%)
N/A	1 (1.5%)	0 (0.0%)	1 (2.3%)
Stage			
I	11 (16.4%)	7 (29.2%)	4 (9.3%)
II	20 (29.9%)	6 (25.0%)	14 (32.6%)
III	18 (26.9%)	1 (4.2%)	17 (39.5%)
IV	18 (26.9%)	10 (41.7%)	8 (18.6%)

NMU: Nanjing Medical University; CCA: cholangiocarcinoma; SD: Standard Deviation; HBV: Hepatitis B virus.

Supplementary Table 2. Summary of exome sequencing results

	NMU		
	Total	iCCA	pCCA
Tumor/normal pair sequenced			
Tumor depth Mean (SD)	94.4 (±11.1)	96.9 (±10.4)	93.0 (±11.3)
Normal depth Mean (SD)	91.8 (±11.5)	94.1 (±11.1)	90.5 (±11.6)
		Pool	
Tumor/normal pair sequenced (mean)			
SNV	84587/112.5	66508/113	18079/112
INDEL	7408/10.5	5329/5	2079/16

NMU: Nanjing Medical University; CCA: cholangiocarcinoma; SNV: Single Nucleotide Variant; INEDL: Insertion and deletion.

Supplementary Table 3. Copy number burden analysis results based on TitanCNA somatic copy numb			
CNV Type	Median CNAB of pCCA	Median CNAB of iCCA	*P**
All Burden	3.91%	17.32%	7.98E-04
Amplification Burden	0.55%	7.50%	1.90E-06
Deletion Burden	1.84%	9.34%	4.34E-02

* *P* value of Wilcoxon rank sum test

Supplementary Table 4. Proportion of COSMIC liver specific signatures

Signature	pCCA		iCCA	
	HBV	non-HBV	HBV	non-HBV
Signature 12	2.03%	0.24%	7.61%	0.10%
Signature 16	3.95%	0.00%	7.76%	3.72%
Signature 24	0.00%	0.00%	10.65%	0.00%
All Signature Liver	5.98%	0.24%	26.02%	3.82%

Supplementary Table 5. Proportion of COSMIC 30 mutation signatures.

Signature	pCCA	iCCA	All
**Signature 1**	**44.71%**	**27.19%**	30.89%
**Signature 6**	**27.52%**	**21.93%**	23.11%
**Signature 4**	**3.73%**	**10.13%**	8.78%
**Signature 22**	**2.37%**	**9.96%**	8.35%
**Signature 15**	**4.47%**	**2.62%**	3.01%
**Signature 9**	**3.05%**	**3.58%**	3.47%
**Signature 8**	**6.41%**	0.00%	1.35%
**Signature 13**	**3.84%**	**1.88%**	2.30%
**Signature 16**	0.00%	**4.50%**	3.55%
**Signature 3**	0.00%	**4.26%**	3.36%
**Signature 2**	**2.64%**	**1.17%**	1.48%
**Signature 12**	0.63%	**2.04%**	1.74%
**Signature 7**	0.44%	**2.17%**	1.81%
**Signature 10**	0.00%	**2.44%**	1.92%
**Signature 26**	0.00%	**2.20%**	1.73%
**Signature 24**	0.00%	**2.16%**	1.71%
Signature 11	0.00%	0.82%	0.65%
Signature 29	0.00%	0.63%	0.50%
Signature 17	0.14%	0.20%	0.19%
Signature 21	0.00%	0.12%	0.10%
Signature 27	0.02%	0.00%	0.00%
Signature 28	0.02%	0.00%	0.00%
Signature 5	0.00%	0.00%	0.00%
Signature 25	0.00%	0.00%	0.00%
Signature 30	0.00%	0.00%	0.00%
Signature 20	0.00%	0.00%	0.00%
Signature 23	0.00%	0.00%	0.00%
Signature 18	0.00%	0.00%	0.00%
Signature 14	0.00%	0.00%	0.00%
Signature 19	0.00%	0.00%	0.00%

Supplementary Table 6. Prognostic effect of copy number burden based on TitanCNA somatic copy number adjusted for tumor purity.

Type	HR	MS L	MS H	*P**
pCCA	2.89	-	404	3.63E-02
iCCA	0.84	600	420	4.52E-01

MS L/H: Median survival time of low/high CNAB subgroup

* *P* value of Cox Proportional-Hazards model adjusted for age, gender, tumor stage and HBV status.

Supplementary Table 7. Curated significantly mutated genes.

Gene	Recurrent mutation	All	iCCA	pCCA	ratio	Subtype specific	class
TP53	1	28.45%	32.18%	17.24%	1.87		Report
KRAS	1	18.39%	19.16%	16.09%	1.19		Report
ARID1A	0	7.18%	8.43%	3.45%	2.44	iCCA	Report
SMAD4	1	6.32%	6.51%	5.75%	1.13		Report
PBRM1	1	5.75%	6.90%	2.30%	3	iCCA	Report
NF1	0	5.75%	4.98%	8.05%	0.62		Report
MACF1	0	5.17%	6.13%	2.30%	2.67	iCCA	New
GNAS	1	5.17%	4.21%	8.05%	0.52		Report
PIK3CA	1	4.89%	5.36%	3.45%	1.56		Report
EPHA2	0	4.60%	5.36%	2.30%	2.33	iCCA	New
BAP1	0	4.60%	4.98%	3.45%	1.44		Report
ARID2	1	4.02%	4.60%	2.30%	2	iCCA	Report
IDH1	1	3.74%	4.98%	0.00%	#DIV/0!	iCCA	Report
ATM	0	3.45%	3.45%	3.45%	1		New
PTEN	1	3.45%	4.21%	1.15%	3.67	iCCA	Report
RBM10	0	3.16%	1.92%	6.90%	0.28	pCCA	New
APC	0	3.16%	3.07%	3.45%	0.89		Report
STK11	0	3.16%	2.68%	4.60%	0.58		Report
RB1	0	2.87%	3.45%	1.15%	3	iCCA	Report
TGFBR2	0	2.87%	2.30%	4.60%	0.5	pCCA	Report
PIK3R1	0	2.59%	1.92%	4.60%	0.42	pCCA	New
BRAF	1	2.59%	3.07%	1.15%	2.67	iCCA	Report
ERBB2	1	2.59%	2.30%	3.45%	0.67		Report
NRAS	1	2.30%	2.68%	1.15%	2.33	iCCA	Report
MLLT4	0	2.01%	2.30%	1.15%	2	iCCA	New
BRCA2	0	2.01%	1.92%	2.30%	0.83		New
SLC8A1	1	2.01%	2.30%	1.15%	2	iCCA	Report
TGFBR1	1	2.01%	1.92%	2.30%	0.83		Report
NACC1	0	1.72%	1.15%	3.45%	0.33	pCCA	New
ELF3	0	1.72%	1.15%	3.45%	0.33	pCCA	Report
SMARCA4	0	1.44%	1.53%	1.15%	1.33		New
WHSC1	0	1.44%	1.53%	1.15%	1.33		New
CTNNB1	1	1.15%	1.15%	1.15%	1		Report
METTL14	1	0.86%	0.38%	2.30%	0.17	pCCA	New
AXIN1	0	0.86%	1.15%	0.00%	#DIV/0!		New
CDKN2A	0	0.86%	0.77%	1.15%	0.67		Report

Supplementary Table 8. Frequently altered focal CNA regions.

	Original Somatic Copy number									Purity-adjusted Somatic Copy number						
	Type	Cytoband	Peak Limits	Gistic2 Q value	CCA	iCCA	pCCA	*P**	Genes	Peak Limits	Gistic2 Q value	CCA	iCCA	pCCA	*P**
Peak 1	Amp	1q21.3	chr1:152884215-153028730	9.98E-05	50.30%	59.52%	23.26%	4.11E-05	*S100A7*	chr1:152136817-152998692	4.08E-04	44.38%	54.76%	13.95%	7.83E-05
Peak 2	Amp	3q29	chr3:195505680-195512202	2.35E-01	27.22%	26.98%	27.91%	1.00E+00	*BCL6/EIF4A2/ETV5/MECOM/LPP/MLF1/MUC4/PIK3CA/SOX2/TFRC/TP63/GMPS/MAP3K13/IGF2BP2/TBL1XR1/MB21D2/MUC4*	chr3:194946409-195800811	6.53E-01	23.08%	23.02%	23.26%	5.46E-01
Peak 3	Amp	5p15.33	chr5:712602-833506	1.01E-01	31.95%	37.30%	16.28%	1.32E-02	*SDHA*	chr5:1094389-3513533	6.47E-01	30.77%	37.30%	11.63%	6.86E-03
Peak 4	Amp	7q31.2	chr7:116201362-117003338	5.23E-03	26.63%	30.95%	13.95%	2.97E-02	*MET*	chr7:115576366-118760185	1.41E-02	27.81%	32.54%	13.95%	2.85E-02
Peak 5	Amp	8q24.21	chr8:127569976-130496004	6.22E-02	51.48%	61.11%	23.26%	1.83E-05	*MYC/NDRG1/FAM135B*	chr8:127272502-129436179	1.82E-06	51.48%	61.11%	23.26%	1.83E-05
Peak 6	Amp	12q15	chr12:69784665-70048359	7.76E-17	24.26%	22.22%	30.23%	3.07E-01	*MDM2*	chr12:68956086-70496201	2.47E-15	22.49%	21.43%	25.58%	2.99E-01
Peak 7	Amp	13q33.3	chr13:108519278-108881202	1.70E-01	18.93%	19.84%	16.28%	8.22E-01	*ERCC5/GPC5/SOX21*	chr13:96120780-115169878	1.50E-01	23.67%	25.40%	18.60%	6.44E-01
Peak 8	Amp	17q12	chr17:37763071-37900028	1.62E-02	22.49%	21.43%	25.58%	6.73E-01	*ERBB2/CDK12*	chr17:37728462-37815781	4.97E-01	19.53%	19.84%	18.60%	5.25E-01
Peak 9	Amp	19q12	chr19:30205961-30934085	5.41E-03	20.71%	22.22%	16.28%	5.15E-01	*CCNE1*	chr19:30985893-41377687	2.34E-02	20.71%	19.05%	25.58%	5.18E-01
Peak 10	Del	1p36.13	chr1:16975100-17019152	8.63E-09	44.97%	47.62%	37.21%	2.88E-01	*SDHB*	chr1:1-29605264	1.15E-10	40.24%	46.03%	23.26%	7.80E-02
Peak 11	Del	2p24.1	chr2:21362860-23608429	1.68E-01	11.83%	9.52%	18.60%	1.68E-01	*DNMT3A/NCOA1/C2orf44/ASXL2*	chr2:1520676-23986790	5.89E-01	8.28%	5.56%	16.28%	4.10E-02
Peak 12	Del	3q29	chr3:195460176-195686566	2.16E-01	10.65%	12.70%	4.65%	1.65E-01	*BCL6/EIF4A2/ETV5/MECOM/LPP/MLF1/MUC4/PIK3CA/SOX2/TFRC/*	chr3:195049593-195800811	6.92E-01	10.06%	11.11%	6.98%	4.11E-01

									TP63/GMPS/MAP3K13/IGF2BP2 / TBL1XR1/MB21D2/MUC4						
Peak 13	Del	4q35.2	chr4:186392465-191154276	4.21E-02	42.60%	49.21%	23.26%	3.97E-03	*CASP3/FAT1*	chr4:163196244-191154276	1.81E-02	36.09%	41.27%	20.93%	3.97E-03
Peak 14	Del	5q14.2	chr5:79852637-81679260	1.34E-01	32.54%	33.33%	30.23%	8.51E-01	*PIK3R1/RAD17*	chr5:64080559-113260791	6.66E-03	31.36%	31.75%	30.23%	8.48E-01
Peak 15	Del	7q35	chr7:143979141-144094614	2.46E-01	11.24%	11.90%	9.30%	7.84E-01	*EZH2/MNX1/FAM131B/ CNTNAP2/KMT2C*			13.02%	12.70%	13.95%	7.85E-01
Peak 16	Del	9p21.3	chr9:21931171-21993843	4.70E-09	51.48%	53.17%	46.51%	4.83E-01	*CDKN2A*	chr9:21942244-22867276	4.23E-02	50.89%	52.38%	46.51%	4.83E-01
Peak 17	Del	10q25.2	chr10:112211448-114192711	1.03E-01	31.95%	34.92%	23.26%	1.87E-01	*FAS/BMPR1A/CYP2C8/FGFR2 /TLX1/MGMT/NFKB2/PTEN/ TCF7L2/CPEB3/NT5C2/SUFU/ VTI1A/KIAA1598/FAM22A*	chr10:89788322-135534747	1.04E-03	28.99%	30.95%	23.26%	8.48E-01
Peak 18	Del	11q23.3	chr11:108811465-116619582	1.85E-01	25.44%	29.37%	13.95%	6.66E-02	*BIRC3/ATM/CBL/DDX6/MLL/ DDX10/FLI1/KCNJ5/PAFAH1B2/ POU2AF1/SDHD/ZBTB16/ ARHGEF12/MAML2/FAT3/ BCL9L/FOXR1*	chr11:97903454-135006516	1.81E-02	22.49%	26.98%	9.30%	6.64E-02
Peak 19	Del	12q24.33	chr12:132213285-133851895	4.67E-03	23.67%	23.81%	23.26%	1.00E+00	*POLE*	chr12:84949118-133851895	2.91E-04	23.08%	24.60%	18.60%	8.35E-01
Peak 20	Del	17p13.3	chr17:1-4017349	1.90E-01	42.60%	42.86%	41.86%	1.00E+00	*PER1/TP53/YWHAE/GAS7/ USP6/RABEP1*	chr17:1-11704306	9.13E-02	43.79%	46.03%	37.21%	1.00E+00
Peak 21	Del	18q21.2	chr18:48190549-49867515	5.18E-02	39.05%	34.92%	51.16%	7.11E-02	*BCL2/DCC/KDSR/SMAD4/MALT 1*	chr18:42889427-64009795	1.12E-05	36.09%	33.33%	44.19%	1.46E-01
Peak 22	Del	19p12	chr19:22379676-22836478	4.89E-02	25.44%	29.37%	13.95%	6.66E-02	*ZNF429*	chr19:20064879-20311900	6.31E-01	41.42%	49.21%	18.60%	6.66E-02

Amp: Amplification; Del: Deletion; *: P value of frequency difference test.

Supplementary Table 9. Curated recurrent mutations

Hugo Symbol	Entrez Gene Id	Chromosome	Start position	End position	Variant Classification	Variant Type	Allele1	Allele2	Sample	cDNA Change	Protein Change	SIFT	CADD	Polyphen2	GERP
ROBO1	6091	3	78766504	78766504	Nonsense Mutation	SNP	C	A	D70036-115	c.838G>T	p.E280*	.	38	.	5.54
ROBO1	6091	3	78766504	78766504	Nonsense Mutation	SNP	C	A	DO222541	c.838G>T	p.E280*	.	38	.	5.54
PTEN	5728	10	89692905	89692905	Missense Mutation	SNP	G	A	D70036-73	c.389G>A	p.R130Q	.	36	0.998	5.22
PTEN	5728	10	89692905	89692905	Missense Mutation	SNP	G	A	DO222603	c.389G>A	p.R130Q	.	36	0.998	5.22
PIK3CA	5290	3	178936091	178936091	Missense Mutation	SNP	G	A	D70036-41	c.1633G>A	p.E545K	0.25	36	0.909	5.78
PIK3CA	5290	3	178936091	178936091	Missense Mutation	SNP	G	A	DO222442	c.1633G>A	p.E545K	0.25	36	0.909	5.78
PIK3CA	5290	3	178936091	178936091	Missense Mutation	SNP	G	A	DO222443	c.1633G>A	p.E545K	0.25	36	0.909	5.78
PIK3CA	5290	3	178936091	178936091	Missense Mutation	SNP	G	A	DO222892	c.1633G>A	p.E545K	0.25	36	0.909	5.78
SPTAN1	6709	9	131378011	131378011	Missense Mutation	SNP	G	A	D70036-37	c.5234G>A	p.R1745H	0.05	35	0.999;0.991;0.995	5.71
SPTAN1	6709	9	131378011	131378011	Missense Mutation	SNP	G	A	DO222955	c.5234G>A	p.R1745H	0.05	35	0.999;0.991;0.995	5.71
VASH2	79805	1	213134569	213134569	Missense Mutation	SNP	C	T	D70036-61	c.338C>T	p.A113V	0.16	34	0.868;0.996;0.994	4.98
METTL14	57721	4	119626803	119626803	Missense Mutation	SNP	G	A	D70036-37	c.893G>A	p.R298H	0.01	28.7	1.0;1.0	4.98
METTL14	57721	4	119626803	119626803	Missense Mutation	SNP	G	A	DO222817	c.893G>A	p.R298H	0.01	28.7	1.0;1.0	4.98
RNF150	57484	4	141868808	141868808	Splice_Site	SNP	C	T	D70036-25	c.890G>A	p.R297Q	.	28.6	0.951;0.726;0.957	5.11
RNF150	57484	4	141868808	141868808	Splice_Site	SNP	C	T	SRR1535216	c.890G>A	p.R297Q	.	28.6	0.951;0.726;0.957	5.11
TGFBR1	7046	9	101891287	101891287	Missense Mutation	SNP	C	T	LD8k0723	c.248C>T	p.P83L	0	28.3	1.0;0.995;0.039	6.08
TGFBR1	7046	9	101891287	101891287	Missense Mutation	SNP	C	T	DO222852	c.248C>T	p.P83L	0	28.3	1.0;0.995;0.039	6.08
NRXN3	9369	14	79181293	79181293	Missense Mutation	SNP	C	T	D70036-37	c.736C>T	p.R246C	.	26.7	0.999;0.886	5.07
NRXN3	9369	14	79181293	79181293	Missense Mutation	SNP	C	T	DO222439	c.736C>T	p.R246C	.	26.7	0.999;0.886	5.07
SEC24C	9632	10	75523303	75523303	Missense Mutation	SNP	G	T	D70036-69	c.1043G>T	p.G348V	0.32	24	0.757;0.644;0.546	5.51
SEC24C	9632	10	75523303	75523303	Missense Mutation	SNP	G	T	DO222740	c.1043G>T	p.G348V	0.32	24	0.757;0.644;0.546	5.51
CTNNB1	1499	3	41266124	41266124	Missense Mutation	SNP	A	G	D70036-87	c.121A>G	p.T41A	0	23.7	0.694	5.91
CTNNB1	1499	3	41266124	41266124	Missense Mutation	SNP	A	G	DO222973	c.121A>G	p.T41A	0	23.7	0.694	5.91
VASH2	79805	1	213134569	213134569	Missense Mutation	SNP	C	A	DO222983	c.338C>A	p.A113E	0.04	23.5	0.987;0.999;0.998	4.98
SLC8A1	6546	2	40656129	40656129	Missense Mutation	SNP	C	T	D70036-49	c.1292G>A	p.R431H	0.01	21.5	0.999;0.999;0.999;1.0;0.999	6.17
SLC8A1	6546	2	40656129	40656129	Missense Mutation	SNP	C	T	DO222952	c.1292G>A	p.R431H	0.01	21.5	0.999;0.999;0.999;1.0;0.999	6.17

ERBB2	2064	17	37879658	37879658	Missense Mutation	SNP	G	A	D70036-41	c.2033G>A	p.R678Q	0.07	20.2	0.103;0.02;0.103	4.97
ERBB2	2064	17	37879658	37879658	Missense Mutation	SNP	G	A	DO222574	c.2033G>A	p.R678Q	0.07	20.2	0.103;0.02;0.103	4.97
ZNF354A	6940	5	178140425	178140425	Frame Shift_Del	DEL	T	-	D70036-87	c.454delA	p.I152fs				
ZNF354A	6940	5	178140425	178140441	Frame Shift_Del	DEL	TTTTTTTGTGGGTGGCT	-	SRR1535082	c.438_454delAGCCACCCACAAAAAAA	p.ATHKKI147fs				
WDTC1	23038	1	27621107	27621108	Frame Shift_Ins	INS	-	G	D70036-87	c.860_861insG	p.MG287fs				
WDTC1	23038	1	27621107	27621108	Frame Shift_Ins	INS	-	G	DO222905	c.860_861insG	p.MG287fs				
WASF3	10810	13	27255387	27255387	Frame Shift_Del	DEL	C	-	D70036-51	c.913delC	p.P310fs				
WASF3	10810	13	27255387	27255387	Frame Shift_Del	DEL	C	-	D70036-87	c.913delC	p.P310fs				
TAP2	6891	6	32805788	32805788	Frame Shift_Del	DEL	G	-	D70036-05	c.223delC	p.L75fs				
TAP2	6891	6	32805788	32805788	Frame Shift_Del	DEL	G	-	D70036-37	c.223delC	p.L75fs				
RPL22	6146	1	6257785	6257785	Frame Shift_Del	DEL	T	-	D70036-05	c.44delA	p.K16fs				
RPL22	6146	1	6257785	6257785	Frame Shift_Del	DEL	T	-	LD8k4455	c.44delA	p.K16fs				
RABL6	55684	9	139734212	139734212	Frame Shift_Del	DEL	C	-	D70036-05	c.1825delC	p.P612fs				
PHLDA1	22822	12	76424936	76424940	Frame Shift_Del	DEL	GTTGC	-	LD8k4465	c.582_586delGCAAC	p.QQQ194fs				
PHLDA1	22822	12	76424936	76424940	Frame Shift_Del	DEL	GTTGC	-	D70036-99	c.582_586delGCAAC	p.QQQ194fs				
LARP4B	23185	10	890939	890939	Frame Shift_Del	DEL	T	-	LD8k4455	c.487delA	p.T163fs				
LARP4B	23185	10	890939	890939	Frame Shift_Del	DEL	T	-	D70036-87	c.487delA	p.T163fs				
KMT2A	4297	11	118344185	118344186	Frame Shift_Ins	INS	-	C	LD8k4455	c.2311_2312insC	p.T771fs				
KMT2A	4297	11	118344185	118344186	Frame Shift_Ins	INS	-	C	DO222427	c.2311_2312insC	p.T771fs				
KLF3	51274	4	38691475	38691476	Frame Shift_Ins	INS	-	C	D70036-99	c.670_671insC	p.S224fs				
KLF3	51274	4	38691475	38691476	Frame Shift_Ins	INS	-	C	DO222913	c.670_671insC	p.S224fs				
GRINA	2907	8	145065478	145065479	Frame Shift_Ins	INS	-	C	DO222454	c.87_88insC	p.P30fs				
DDX5	1655	17	62500099	62500102	Splice Site	DEL	ACAG	-	D70036-87	c.440_442delCTGT	p.SV147fs				
DDX5	1655	17	62500099	62500099	Splice Site	ONP	ACAG	ACAG	DO222955						
CCDC116	164592	22	21989446	21989446	Frame Shift_Del	DEL	C	-	LD8k4455	c.1094delC	p.S365fs				
CCDC116	164592	22	21989446	21989446	Frame Shift_Del	DEL	C	-	D70036-87	c.1094delC	p.S365fs				

BCL9	607	1	147091500	147091501	Frame Shift_Ins	INS	-	C	LD8k4455	c.1539_1540insC	p.P514fs
BCL9	607	1	147091500	147091501	Frame Shift_Ins	INS	-	CC	DO222427	c.1539_1540insCC	p.P514fs
ATP8B1	5205	18	55365039	55365040	Frame Shift_Ins	INS	-	T	D70036-05	c.614_615insA	p.N205fs
ATP8B1	5205	18	55365039	55365040	Frame Shift_Ins	INS	-	T	D70036-99	c.614_615insA	p.N205fs
ATF7IP	55729	12	14577801	14577802	Frame Shift_Ins	INS	-	A	D70036-05	c.952_953insA	p.E318fs
ATF7IP	55729	12	14577801	14577802	Frame Shift_Ins	INS	-	A	D70036-99	c.952_953insA	p.E318fs

SNP: single nucleotide polymorphism; DEL: Deletion; INS: Insertion; SIFT: Sorting Intolerant from Tolerant; CADD: Combined Annotation-Dependent Depletion; GERP: Genomic Evolutionary Rate Profiling
