# Supplementary material for: The genomic landscape of cholangiocarcinoma reveals the disruption of post-transcriptional modifiers
Source: Nat Commun. 2022 Jun 1;13:3061. doi: 10.1038/s41467-022-30708-7 (PMC9160072; doi:10.1038/s41467-022-30708-7)
Supplement: Supplementary file 3 — Reporting Summary [file 41467_2022_30708_MOESM3_ESM.pdf]

nature portfolio

Corresponding author(s): Xiangcheng Li

Last updated by author(s): Jan 15, 2022

Reporting Summary

Nature Portfolio wishes to improve the reproducibility of the work that we publish. This form provides structure for consistency and transparency in reporting. For further information on Nature Portfolio policies, see our Editorial Policies and the Editorial Policy Checklist.

Statistics

For all statistical analyses, confirm that the following items are present in the figure legend, table legend, main text, or Methods section.

n/a	Confirmed	
☐	☒	The exact sample size (n) for each experimental group/condition, given as a discrete number and unit of measurement
☐	☒	A statement on whether measurements were taken from distinct samples or whether the same sample was measured repeatedly
☐	☒	The statistical test(s) used AND whether they are one- or two-sided<br>*Only common tests should be described solely by name; describe more complex techniques in the Methods section.*
☒	☐	A description of all covariates tested
☒	☐	A description of any assumptions or corrections, such as tests of normality and adjustment for multiple comparisons
☐	☒	A full description of the statistical parameters including central tendency (e.g. means) or other basic estimates (e.g. regression coefficient) AND variation (e.g. standard deviation) or associated estimates of uncertainty (e.g. confidence intervals)
☐	☒	For null hypothesis testing, the test statistic (e.g. F, t, r) with confidence intervals, effect sizes, degrees of freedom and P value noted<br>*Give P values as exact values whenever suitable.*
☒	☐	For Bayesian analysis, information on the choice of priors and Markov chain Monte Carlo settings
☒	☐	For hierarchical and complex designs, identification of the appropriate level for tests and full reporting of outcomes
☒	☐	Estimates of effect sizes (e.g. Cohen's d, Pearson's r), indicating how they were calculated

Our web collection on statistics for biologists contains articles on many of the points above.

Software and code

Policy information about availability of computer code

Data collection	Publicly available datasets were obtained and used from Zou et al's study, which we downloaded the raw FASTQ files from Short Read Archive (SRA) under the accession code SRP045202 and GSE2150. For integration analysis, we applied the somatic mutation data of 239 patients with cancer of biliary duct, including 44 pCCAs and 135 iCCAs, from BTCA-JP, International Cancer Genome Consortium (ICGC) project (https://icgc.org/icgc/cgp/91/420/1012366).<br>Library construction and whole-exome capture of genomic DNA were performed using the Roche NimbleGen SeqCap EZ Exome SR platform V3. The captured DNA was sequenced on an Illumina HiSeq X10 sequencing system, with 150-bp paired-end sequencing.<br>Detailed information about clinical sample collection; Next-generation sequencing; Sequencing alignment and detection of somatic variants; Mutational burden and signature analysis; Identification of CCA potential driver genes and comparison between the iCCA and pCCA; Somatic copy number estimation; Highly amplified/deleted regions identification; Pathway enrichment analysis; Annotation of genomic alterations upon clinical actionability are available in the supplementary material.
Data analysis	The median was used if multiple samples from the same tissues were sequenced. All statistical tests were performed using a Wilcoxon rank-sum test for continuous data. Fisher's exact test was used to assess differences in the count data. Multiple testing corrections were performed where necessary using the Benjamini-Hochberg method. All reported P values were two-sided. Mutational lolliplots were generated by ProteinPaint.

For manuscripts utilizing custom algorithms or software that are central to the research but not yet described in published literature, software must be made available to editors and reviewers. We strongly encourage code deposition in a community repository (e.g. GitHub). See the Nature Portfolio guidelines for submitting code & software for further information.

March 2021

Data

Policy information about availability of data

All manuscripts must include a data availability statement. This statement should provide the following information, where applicable:

- Accession codes, unique identifiers, or web links for publicly available datasets
- A description of any restrictions on data availability
- For clinical datasets or third party data, please ensure that the statement adheres to our policy

Data Availability
The WES (accession number: HRA001570[https://bigd.big.ac.cn/gsa-human/browse/HRA001570]) and RNA m6A sequencing (accession number: HRA001826[https://bigd.big.ac.cn/gsa-human/browse/HRA001826]) raw data of NMU patients with pCCA or iCCA have been deposited in Genome Sequence Archive (GSA) for human hosted by China National Center for Bioinformation (CNCB) under BioProject PRJCA006646 [https://ngdc.cncb.ac.cn/bioproject/browse/PRJCA006646]. The publicly available WES raw data obtained from Zou et al's study were downloaded from Short Read Archive (SRA) under the accession code SRP045202 [https://trace.ncbi.nlm.nih.gov/Traces/sra/?study=SRP045202]. The raw data of BTCA-JP were obtained from International Cancer Genome Consortium (ICGC) project [https://dcc.icgc.org/projects/BTCA-JP]. The authors declare that the source data supporting the findings of this study are available from the corresponding author upon request. The applicant and his/her institute need to get approved by the MOST of the People's Republic of China following Regulation of the People's Republic of China on the Administration of Human Genetic Resources.

Field-specific reporting

Please select the one below that is the best fit for your research. If you are not sure, read the appropriate sections before making your selection.

☒ Life sciences ☐ Behavioural & social sciences ☐ Ecological, evolutionary & environmental sciences

For a reference copy of the document with all sections, see nature.com/documents/nr-reporting-summary-flat.pdf

Life sciences study design

All studies must disclose on these points even when the disclosure is negative.

Sample size	No sample size calculation was performed. Sample size was determined based on the level and consistency between two different groups. All data sets include at least three biological replicates.
Data exclusions	No data were excluded from analysis.
Replication	All experimental data are given including replicates. Details of experimental replicates are given in the figure legends. All reported attempts at replication were successful.
Randomization	All allocations were random in this study
Blinding	All data collection and analysis was blinded.

Reporting for specific materials, systems and methods

We require information from authors about some types of materials, experimental systems and methods used in many studies. Here, indicate whether each material, system or method listed is relevant to your study. If you are not sure if a list item applies to your research, read the appropriate section before selecting a response.

Materials & experimental systems

n/a	Involved in the study	
☐	☒	Antibodies
☐	☒	Eukaryotic cell lines
☒	☐	Palaeontology and archaeology
☐	☒	Animals and other organisms
☐	☒	Human research participants
☒	☐	Clinical data
☒	☐	Dual use research of concern

Methods

n/a	Involved in the study	
☒	☐	ChIP-seq
☐	☒	Flow cytometry
☒	☐	MRI-based neuroimaging

Antibodies

Antibodies used	METTL14, Novus, cat.no: NBP1-81392, Clone no: Polyclonal; WB 1/1000 IHC 1/1000 IF 1/1000<br>METTL14, Proteintech, cat.no: 26158-1-AP, Clone no: Polyclonal; RIP 1/100<br>GAPDH, Abcam, cat.no: ab181602 monoclonal, Clone no: EPR16891; WB 1/2500

Lamin B1 Proteintech, cat.no: 12987-1-AP, Clone no: Polyclonal; WB 1/500
β-catenin, Abcam, cat.no: ab32572, Clone no: monoclonal [E247]; WB 1/1000
E-cadherin, Cell Signaling Technology,cat.no: #3195, Clone no: 24E10; WB 1/1000
N-cadherin, Cell Signaling Technology, cat.no: #13116, Clone no: D4R1H; WB 1/1000
α-Tubulin, Cell Signaling Technology,cat.no: #2125, Clone no: 11H10; WB 1/1000
PCNA, Abcam, cat.no: ab92552, Clone no: EPR3821; WB 1/1000
Cyclin D1, Cell Signaling Technology, cat.no: #2922, Clone no: Polyclonal; WB 1/1000
ATP1A1, Cell Signaling Technology, cat.no: #23565, Clone no: D4Y7E; WB 1/1000
MACF1, Abcam, cat.no: ab221989, Clone no: Polyclonal; IF 1/300
MACF1, Proteintech, cat.no: 13058-1-AP, Clone no: Polyclonal; WB 1/500
M6A-specific antibody, Synaptic Systems, cat.no: No. 202003; Clone no: Polyclonal;RIP 1/200

Validation

METTL14(NBP1-81392): Winkler R, Gillis E, Lasman L et al. m6A modification controls the innate immune response to infection by targeting type I interferons Nat. Immunol. Dec 17 2018;
GAPDH (ab181602): Rong Y et al. The Golgi microtubules regulate single cell durotaxis. EMBO Rep 22: e51094 (2021);
Lamin B1(12987-1-AP): Li B, Cao Y, Meng G et al. Targeting glutaminase 1 attenuates stemness properties in hepatocellular carcinoma by increasing reactive oxygen species and suppressing Wnt/beta-catenin pathway. EBioMedicine. 2019 Jan;39:239-254.
β-catenin(ab32572): Yang D et al. WNT4 secreted by tumor tissues promotes tumor progression in colorectal cancer by activation of the Wnt/ß-catenin signalling pathway. J Exp Clin Cancer Res 39:251
E-cadherin (#3195): Xi He, et. al.Tumor-initiating stem cell shapes its microenvironment into an immunosuppressive barrier and pro-tumorigenic niche. 2021
N-cadherin (#13116): Stancil IT et al. Pulmonary fibrosis distal airway epithelia are dynamically and structurally dysfunctional. Nat Commun. 2021 Jul 27;12(1):4566.
α-Tubulin (#2125): Du Y et al. A broadly neutralizing humanized ACE2-targeting antibody against SARS-CoV-2 variants. Nat Commun. 2021 Aug 17;12(1):5000.
PCNA (ab92552): Zierold S et al. Brain-Derived Neurotrophic Factor Expression and Signaling in Different Perivascular Adipose Tissue Depots of Patients With Coronary Artery Disease. J Am Heart Assoc 10:e018322 (2021).
Cyclin D1 (#2922): Gong FX et al. De-dimerization of PTB is catalyzed by PDI and is involved in the regulation of p53 translation. Nucleic Acids Res. 2021 Sep 20;49(16):9342-9352.
MACF1 (ab221989): AbpromiseTM guarantees the use of ab221989 in immunofluorescence experiments
MACF1 (13058-1-AP): Hu L et al. MACF1 promotes osteoblast differentiation by sequestering repressors in cytoplasm. Cell Death Differ. 2021 Jul;28(7):2160-2178. Epub 2021 Mar 4.
M6A-specific antibody (202003): Xie Q et al. N6-methyladenine DNA Modification in Glioblastoma. Cell (2018)

Eukaryotic cell lines

Policy information about cell lines

Cell line source(s)

RBE, Supplier: Cell Bank of the Chinese Academy of Science, Cat no.TCHu179,
HCCC9810, Supplier: Cell Bank of the Chinese Academy of Science, Cat no.TCHu 17, Authentication test method: Cell Bank of the Chinese Academy of Science

Authentication

The lines were authenticated by short tandem repeat (STR) profiling.

Mycoplasma contamination

ALL CELLINES USED WERE MYCOPLASMA FREE

Commonly misidentified lines
(See ICLAC register)

None as far as we know

Animals and other organisms

Policy information about studies involving animals; ARRIVE guidelines recommended for reporting animal research

Laboratory animals

4-week-old BALB/c nude male mice for the subcutaneous tumor growth assay, 6-week-old male nude mice for the lung metastasis experiment. Mice were housed in specific pathogen free (SPF) conditions, dark/light cycles: 12-hours light/12-hour dark (150-300 lux), ambient temperature 20-26℃ degrees Celsius℃ and humidity 40%-70%, ventilated four times per hour

Wild animals

We did not use any wild animals.

Field-collected samples

Studies did not include samples collected from the field.

Ethics oversight

All the animal studies were approved by the Institutional Animal Care and Use Committee of Nanjing Medical University and conducted according to protocols approved by the Ethical Committee of Nanjing Medical university.

Note that full information on the approval of the study protocol must also be provided in the manuscript.

Human research participants

Policy information about studies involving human research participants

Population characteristics	All primary cholangiocarcinoma and matched adjacent normal samples were obtained from the resected specimens of patients with pCCA or iCCA between 2010 and 2017 in The Affiliated Hospital of Nanjing Medical University (NMU).
Recruitment	The use of clinical samples was approved by the Ethics Committee of The Affiliated Hospital of Nanjing Medical University. Written informed patient consent was obtained in accordance with regional regulation. The data of their clinicopathological features were anonymized. All tumor samples were confirmed by pathologists that there was a minimum tumor cellularity of 70% in all CCA specimens following histopathological review of H&E slides.
Ethics oversight	The use of clinical samples was approved by the Ethics Committee of The Affiliated Hospital of Nanjing Medical University. Written informed patient consent was obtained in accordance with regional regulation. The data of their clinicopathological features were anonymized.

Note that full information on the approval of the study protocol must also be provided in the manuscript.

Flow Cytometry

Plots

Confirm that:

☒ The axis labels state the marker and fluorochrome used (e.g. CD4-FITC).

☒ The axis scales are clearly visible. Include numbers along axes only for bottom left plot of group (a 'group' is an analysis of identical markers).

☒ All plots are contour plots with outliers or pseudocolor plots.

☒ A numerical value for number of cells or percentage (with statistics) is provided.

Methodology

Sample preparation	The cell apoptosis was monitored by Annexin V/PI apoptosis kit (Multisciences, Hangzhou, China). Cells seeded in 6-well plates were treated with 5μl/ml 0.3% hydrogen peroxide. Cells were then stained with Annexin V/PI binding buffer for 5 minutes protected from light.
Instrument	BD FACS AriaIII
Software	Data analysis was performed in FlowJo (V10).
Cell population abundance	Percentage of early, late apoptotic cells were quantified with FlowJo V10 according to the manufacturer's instructions.
Gating strategy	Whole cells were gated using FSC and SSC, and alive cells were classified as Annexin V-/PI. The gating strategy is also provided in the Supplement Information.

☒ Tick this box to confirm that a figure exemplifying the gating strategy is provided in the Supplementary Information.
